# Predictors of Candidemia during Febrile Episode in Lymphoreticular Malignancy Affecting Paediatric Population

**DOI:** 10.3390/diagnostics13091638

**Published:** 2023-05-05

**Authors:** Suchita Gautam, Shukla Das, Praveen Kumar Singh, Gargi Rai, Charu Jain, Rumpa Saha, Narendra Pal Singh, Sunil Gomber, Raga Eltayeb, Sajad Ahmad Dar

**Affiliations:** 1 Department of Microbiology, University College of Medical Sciences (University of Delhi) & GTB Hospital, Delhi 110095, Indiadoccharujain@gmail.com (C.J.);; 2 Department of Paediatrics, University College of Medical Sciences (University of Delhi) & GTB Hospital, Delhi 110095, India; 3 Department of Pathology, College of Medicine, Jazan University, Jazan 45142, Saudi Arabia; 4 Research and Scientific Studies Unit, College of Nursing, Jazan University, Jazan 45142, Saudi Arabia

**Keywords:** candidemia, colonization index, febrile episode, lymphoreticular malignancy, mannan antigen

## Abstract

Limited studies on candidemia in malignancy in the paediatric population from developing countries show a high incidence, high morbidity and a unique epidemiology as compared to developed nations. Our prospective observational study aimed to explore the prevalence of invasive candidiasis, especially candidemia, in febrile paediatric patients with lymphoreticular malignancy. A sample size of 49 children, with 100 recorded febrile episodes was studied. The relevance of candida colonization and mannan antigen detection as indicators of impending candidemia was evaluated. Genotypic identification of the yeast isolates was followed by sequence analysis using the NCBI-BLAST program, and the generation of the phylogenetic tree using MEGA 6.0 software. We observed a 5% prevalence of candidemia among febrile paediatric patients with lymphoreticular malignancy, predominantly caused by non-albicans candida. Colonization at multiple anatomical sites decreased from day 1 to day 8 of febrile episodes. Significant candida colonization (colonization index ≥0.5) was seen in a larger proportion of candidemia patients on day 1 and day 4 (*p* < 0.001) displaying a definite association between the two. The receiver operator characteristic (ROC) curve analysis for mannan antigen level revealed a cut-off of ≥104.667 pg/mL, suitable for predicting candidemia with a sensitivity of 100%, specificity of 92% and area under ROC value of 0.958 (95% CI: 0.915–1; *p* < 0.001). A phylogenetic tree with three population groups, clade 1, 2 and 3, consisting of *Candida auris* (1), *Candida tropicalis* (2) and *Candida parapsilosis* (2), respectively, was generated. The diagnosis of candidemia based on mannan antigen detection gives early results and has high negative predictive values. It can be combined with other biomarkers to increase sensitivity, specificity and positive predictive value.

## 1. Introduction

Fever is a non-specific but frequently the only symptom of infection in children with lymphoreticular malignancy receiving chemotherapy. Other factors such as neutropenia, immunosuppressive therapy and poor immune response makes this group more susceptible to acquire infection. Febrile neutropenia (FN) is a fever associated with abnormally low neutrophil count signifying an immune-compromised state secondary to malignancy or its treatment. It is a common and often critical condition that adversely impacts the prognosis of patients. FN is defined as the single oral temperature above 38 °C (101 °F) over 1 h with <500 neutrophils/mm^3^ or <1000 neutrophils/mm^3^ with a predicted decline to 500/mm^3^ over the next 48 h [1]. In the setting of cytotoxic drug-induced neutropenia, the colonizing yeast may penetrate the damaged mucosal barriers and invade the blood stream [2]. Candida and aspergillus species account for 75% of fungal infection and 25–60% of mortality [3].

Data on candidemia in malignancy in the paediatric population is mainly available from developed nations of North America and Western Europe, however, only a few epidemiological studies are available from developing countries of Asia, Africa and Latin America, constituting 80% of the world’s population. Limited studies from developing nations shows a high incidence, high morbidity and unique epidemiology as compared to developed nations. A study from South India showed the incidence of candidemia as 5.7% among such children [3]. Most of data available on candidemia is from ICUs or general hospital admissions [4].

The present study was planned to explore the prevalence of invasive candidiasis, especially candidemia, in febrile paediatric patients with lymphoreticular malignancy. We evaluated the relevance of Candida colonization and mannan antigen detection as indicators of impending candidemia.

## 2. Materials and Methods

### 2.1. Patient Selection

Our study was a prospective observational study conducted for 18 months, from November 2018 to April 2020 at the paediatric Oncology Unit of a multispecialty tertiary care centre. Based on our patient load, the duration of the proposed study, and available resources, a sample size of 49 (*n* = 49) was decided and 100 febrile episodes in children with lymphoreticular malignancy were studied. Children below 12 years, receiving chemotherapy for haematological malignancy having oral or axillary temperature more than 38.3 °C for more than one hour were included in this study. Children receiving antifungal treatment in last 7 days were excluded from the study. Routine blood investigations (CBC with absolute neutrophil count) were performed on day 1 and 4 and a blood culture/sensitivity for bacterial and fungal pathogens in BACTEC 9120 system was performed on day 1 and day 4 of each febrile episode. The study was approved by the Institutional Ethical Committee—Human Research (IEC-HR) of the institute and written informed consent was obtained from parents/legal guardians of the children prior to starting the study.

### 2.2. Assessing Candida Colonization and Antifungal Susceptibility

Sputum, pharyngeal swab, umbilical swab, axillary swab, rectal swabs and urine were collected on day 1, day 4 and day 8 and followed up for each febrile case. All yeast isolates were subjected to Gram’s stain, germ tube test followed by identification on MICROSCAN (from Beckman Coulter). The yeast isolates were subjected to antifungal susceptibility testing using the commercial E-test strips (Hi-Media, Mumbai, India) for fluconazole (0.016–256 mcg/mL), Amphotericin-B (0.002–32 mcg/mL) and caspofungin (0.002–32 mcg/mL). Interpretive guidelines for in vitro susceptibility of *Candida* spp. (M27-S3 and S4) was followed to determine the susceptibility of isolates to different antifungals.

Based on colonization index on day 1, day 4 and day 8, candida colonization was evaluated among all febrile patients. The colonization index was defined as the ratio of the number of distinct body sites colonized by *Candida* spp. to the total number of body sites swabbed and cultured. A colonization index value ≥0.5 was considered as the threshold for significant candida colonization [5].

### 2.3. Mannan Antigen Detection

Venous blood of 3 mL was collected from all patients with febrile episodes on day 1 for serum mannan antigen detection by commercial kit (Platelia™ Candida Ag EIA; Bio-Rad, Marnes-la-Coquette, France). Samples with concentrations equal or greater than 125 pg/mL were considered to be positive for mannan antigen as stated in the manufacturer’s instructions. Briefly, the patient samples, negative control (R0) and positive control (R5) were treated at the same time, all by adding 100 μL of treatment solution (R7). Ready to use calibrators—R3, R4a, R4b, R4c and R4d—were not treated. Three hundred (300) μL of each patient sample, of the negative control (R0) and of the positive control (R5) was pipetted into individual 1.5 mL polypropylene tube. One hundred (100) μL of the sample treatment solution (R7) was added to each tube. Tubes were mixed thoroughly by vigorous mixing or vortexing. Tubes were closed to prevent opening during heating. The tubes were heated for 3 min at 100 °C in boiling water bath. The hot tubes were removed from the boiling water bath and centrifuged at 10,000 g for 10 min. The supernatant was used for the detection of the mannan antigen. Samples with concentrations less than 62.5 pg/mL (C < 62.5) were considered to be ‘negative’ for mannan antigen. Samples with concentrations between 62.5 and 125 pg/mL (62.5 ≤ C < 125) were considered to be ‘intermediate’ for mannan antigen. Samples with concentrations that are equal or greater than 125 pg/mL (C ≥ 125) were considered to be ‘positive’ for mannan antigen.

### 2.4. Genotypic Identification of the Yeast Isolates

DNA extraction was done from yeast colonies grown in SDA medium, using commercially available DNA extraction kit (Hi-Yield Genomic DNA kit, RBC Germany). Genomic DNA was subjected to PCR amplification using pan-fungal internal transcribed spacer (ITS) region primers:

ITS1: 5′-TCCGTAGGTGAACCTGCGG-3′
ITS4: 3′-TCCTCCGCTTATTGATATGC-5′

The PCR amplicons were resolved on 2% agarose gel with 0.5 mM ethidium bromide and visualized on UV transilluminator.

Sequencing analysis of ITS gene region was evaluated by using the National Centre for Biotechnology information (NCBI, Bethesda, MD, USA) BLAST system “http://www.ncbi.nlm.nih.gov/blast (accessed on 10 March 2023)”. The sequencing performed using ITS1 and ITS4 regions were compared with sequences deposited in GeneBank by using the Blast program to find identical or similar sequences.

### 2.5. Phylogenetic Analysis

Sequenced nucleotides with multiple alignments were carried out using Clustal W2 (version 2.0.10) and the neighbour-joining method to construct the tree by MEGA 6.0 [6]. A bootstrap procedure with 1000 replicates was used to check node consistency. A phylogenetic tree was generated using sequences from five isolates and sequences retrieved from gene bank.

### 2.6. Statistical Analysis

Data was coded and recorded in a MS Excel spread sheet. *SPSS* was used for data analysis. Group comparisons for continuously distributed data were made using independent sample *t*-test when comparing two groups. If data were not in normal distribution, appropriate non-parametric tests in the form of Wilcoxon tests were used. The chi-squared test was used for group comparisons for categorical data. In case the expected frequency in the contingency tables was found to be <5 for >25% of the cells, Fisher’s exact test was used instead.

## 3. Results

### 3.1. Febrile Episodes in Patients

Out of the total 49 child patients recruited in this prospective observational study, 40 were males and 9 were females. The type of cancer observed in these patients were 42 B-cell acute lymphoblastic leukaemia (ALL), four T-cell ALL, one acute myeloid leukemia (AML), and one each of Hodgkin’s lymphoma (HL) and non-Hodgkin’s lymphoma (NHL). Out of 100 febrile episodes, candidemia was present in five episodes of five different patients. There were multiple admissions of these patients during the course of the study. Only single febrile episode was considered in one admission, therefore total number of febrile episodes was considered as total number of cases during study period. Seventy-seven episodes occurred in males and 23 in females. Multiple numbers of febrile cases were recorded in a single patient which varied from one episode to a maximum five episodes per patients. Thirty-three febrile episodes occurred in age less than 5 years, 50 episodes occurred between 5 and 10 years and 17 episodes occurred in age more than or equals to 10 years. Out of 100 febrile episodes recorded, 84 episodes occurred in patients with B-cell ALL, 10 episodes in T-cell ALL, four episodes in AML, and one episode each in HL and NHL.

### 3.2. Pathogens Isolated from Blood Culture

Six percent (6%) of the 100 blood cultures processed on day 1 of febrile episodes were positive for bacteria. Eighteen percent (18%) of blood cultures were positive when collected on day 4, of which *Candida* spp. was isolated from five percent (5%). Out of five *Candida* spp., two (40%) isolates of *C. parapsilosis*, two (40%) isolates of *C. tropicalis* and one (20%) of *C. auris* were recovered. All of the candidemia cases (5 out of 100 cases), flagged candida positive were observed on day 4 of febrile episodes. Out of five cases of candidemia, three cases occurred in patients with B-cell ALL, one case in T-cell ALL and one in AML.

Amongst the bacteria species isolated on day 1 and day 4, four were coagulase negative *Staphylococci*, two were *Staphylococcus aureus*, two were methicillin resistant *Staphylococcus aureus*, four were *Escherichia coli*, and two were *Citrobacter freundii*.

### 3.3. Clinical Parameters Associated with Candidemia

Clinical parameters were compared between two groups of patients with and without candidemia but with associated febrile episodes. Sixty one percent (61%) of patients had only fever with no associated symptoms. Gastrointestinal symptoms such as pain abdomen and loose stools were the other common complaints. Abdominal pain was found to be significantly associated with presence of candidemia (Table 1).

A significant association of candidemia with low total leucocyte count (TLC) and absolute neutrophil count (ANC) was observed at day 4 compared to febrile episodes without candidemia (Table 1).

### 3.4. Candida Colonization among Patients

Significant colonization was observed in episodes of candidemia. Four (80%) cases on day 1, 3 (60%) cases on day 4 and 1 (20%) case on day 8 had significant colonization among the patient who were diagnosed with candidemia on follow up, while only one (1.05%) case had significant colonization on both day 1 and day 4 in non-candidemic patients (Table 2). Candida colonization among all patients from various anatomical sites had decreased over time. Candida colonization was highest in pharyngeal/oral swab of the patients (Table 3). A total of five of the febrile cases on day 1 and four on day 4 showed significant colonization (colonization index ≥ 0.5) as shown in Table 2.

*Candida albicans* was the most common colonizing species followed by *C. parapsilosis* and *C. tropicalis.* Candida species commonly isolated on blood culture during our study were *C. parapsilosis*, *C. tropicalis* and *C. auris* which were most commonly colonizing in oropharynx, urinary tract and rectum suggesting possible endogenous spread (Table 3).

Antifungal susceptibility of all Candida spp. isolated from blood as evaluated by E-strip method showed 100% sensitive to fluconazole Amphotericin-B and caspofungin.

### 3.5. Mannan Antigen Serology and Receiver Operator Characteristic (ROC) Curve

**Figure 1:**
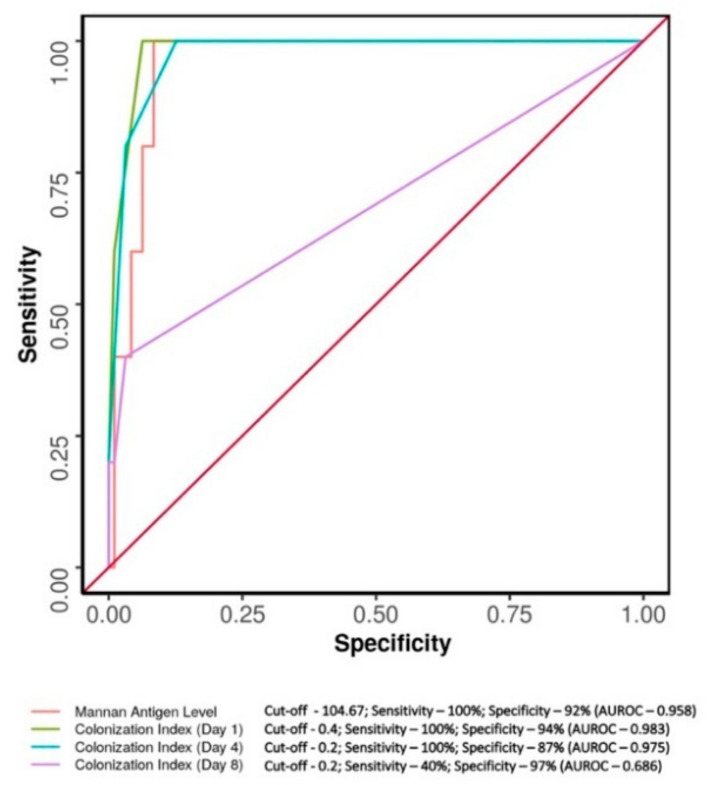
Comparison of the diagnostic performance of various parameters in predicting candidemia. ROC curve analysis showing diagnostic performance of colonization index (for day 1, 4 and 8), and mannan antigen level in predicting candidemia.

Serology evaluation showed 11% of febrile episodes were positive and 89% of episodes were negative for mannan antigen. The ROC curve analysis, for assessing diagnostic performance in predicting candidemia, of mannan antigen and colonization index (for different days) at different cut-off values was performed (Figure 1 and Table 4). The colonization index (day 1) at a cut-off of ≥0.4 had a sensitivity of 100%, and a specificity of 94% with AUROC equal to 0.983 (95% CI: 0.961–1; *p* < 0.001). The colonization index (day 4) at a cut-off of ≥0.2 had sensitivity of 100%, and a specificity of 87% with AUROC equal to 0.975 (95% CI: 0.941–1; *p* < 0.001). The ROC analysis for mannan antigen level revealed a cut-off of ≥104.667 pg/mL, having a sensitivity of 100%, and a specificity of 92% with AUROC equal to 0.958 (95% CI: 0.915–1; *p* = 0.001).

Colonization index on day 1 and 4 have good negative predictive values (NPV) with high diagnostic accuracy. ROC for colonization index on day 1 and 4 showed 100% NPV at the cut-off of 0.4 and 0.2, respectively (Table 5).

The association of candidemia with candida mannan antigen detection is statistically analysed and highlighted in Table 6. As per the analyses, different primary diagnostic parameters were ranked and colonization index at day 1 (cut-off of 0.4 by ROC) was best to rule out candidemia with moderate positive predictive value (Table 7).

### 3.6. Genotypic Identification and Phylogenetic Analysis

**Figure 2:**
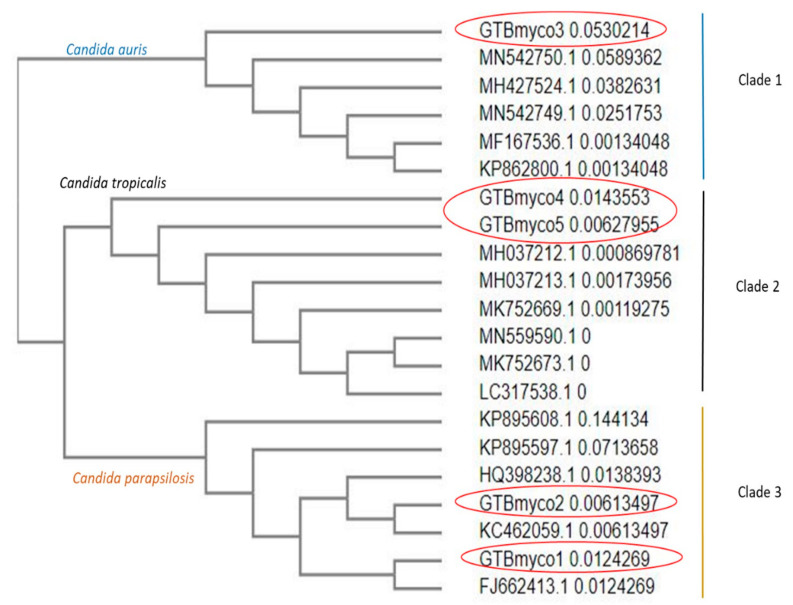
Phylogenetic analysis: we compared sequences of our candida isolates to those deposited in the GenBank to determine their intraspecific variability. The possible formation of three population groups—Clade 1, 2, 3, consisting of the one isolate of *Candida auris*; two isolates of *Candida tropicalis*; two isolates of *Candida parapsilosis*, respectively, were observed.

The ITS sequences displayed >99% similarity with specific *Candida* spp. On phylogenetic analysis we observed the possible formation of three population groups—Clade 1, 2, 3 consisting of one isolate of *C. auris*; two isolates of *C. tropicalis* and two isolates of *C. parapsilosis*, respectively (Figure 2).

## 4. Discussion

Candidemia is the most important cause of invasive fungal infections in hospitalized children. Children with lymphoreticular malignancy are highly susceptible to acquire infection because of neutropenia secondary to antineoplastic drug therapy. In the setting of cytotoxic drug induced neutropenia, the colonizing yeast penetrate the damaged mucosal barriers and invade blood stream. Hence, considering the potential risk of acquiring fungal infection in these patients, early detection and prompt treatment of candidemia becomes essential. Timely antifungal therapy and source control are crucial determining factors of survival in patients. Definitive treatment is often delayed due to the high turn over time and limited sensitivity of blood culture, the gold standard diagnostic technique. Limited studies from developing nations reported high incidence, high morbidity and unique epidemiology due to candida infection as compared to developed nations. Additionally, candida mannan antigen detection is a valuable serological marker for candidemia.

The prevalence of candidemia among febrile paediatric patients with lymphoreticular malignancy was 5% in this study. In our study, none of the patients were on central venous catheter or prior use of TPN and had no history of any abdominal surgery. Candida colonization was a risk factor for candidemia and serum mannan antigen was a good immunological marker for detection of candidemia during the febrile episodes in children with malignancy. There are very few studies reporting the prevalence of candidemia in paediatric malignancies in India. Depending on the patient profile studied, geographical location and diagnostic criteria used, the prevalence of candidemia among patients with haematological malignancies has been found to vary between 1.6% and 22.9% [7,8,9]. Most of the studies from Indian centres have reported bacterial infections occurring in haematological malignancies [10,11], but few studies have documented the epidemiology and mycological characteristic of candidemia. A study reported prevalence of candidemia among paediatric patients with haematological malignancy as 5.7% in south India [3]. They reported the isolation of *Candida tropicalis* and *Candida albicans* from blood. A world-wide shift has been noted in the species responsible for candidemia. The shift has been partially attributed to the excessive use of azoles prophylaxis. Children and young adults <20 years old showed *C. tropicalis* as the common pathogen associated with candidemia in neutropenic patients with hematologic malignancies [12]. Though lower prevalence (5%) was observed in our study compared to other Indian studies [13], the predominance of non-albicans candida species was undoubtedly evident from our blood culture analysis.

We observed colonization at multiple anatomical sites decreased from day 1 to day 8 of febrile episodes. Significant colonization (CI ≥ 0.5) was seen in a larger proportion of candidemia patients on day 1 and day 4 (*p* < 0.001) displaying a definite association between candida colonization and occurrence of candidemia. The timely administration of antifungal prophylaxis seems to be a probable cause of the decrease in the rate of colonization from day 1 to day 8. Surprisingly, *C. albicans* was the most frequent colonizing species and oropharynx was most common site of colonization. However, C. *parapsilosis*, *C. tropicalis* and *C. auris*, as isolated from blood, were found to be colonizing most commonly the oropharynx, urinary tract and rectum, suggesting their possible role in endogenous gut transmigration causing blood stream candida infection.

Several colonization studies have documented the association of multiple site colonization with high risk of candidemia among cancer patients and have noted a definite shift from *C. albicans* to non-albicans *Candida* species [14,15,16,17,18,19,20,21,22]. The principle of colonization index with threshold value CI ≥ 0.5 has been widely explored in critically ill patients admitted in ICU or in those who have underdone abdominal surgery [5,23,24]. We used the same threshold value to explore the association between colonization and candidemia in febrile paediatric patients with lymphoreticular malignancy and found that colonization is considered a cardinal risk factor for candidemia in febrile paediatric patients with lymphoreticular malignancies when diagnosed on day 1 up to day 4 of febrile episode. Interestingly, colonization at umbilicus and axilla were not associated with candidemia.

There are limited studies regarding colonization index threshold values to be used in febrile paediatrics patients with lymphoreticular malignancy. Colonization index day 1 and mannan antigen level have good negative predictive values with high diagnostic accuracy. In our study the diagnostic performance of colonization index at the observed cut-off of CI ≥ 0.4 was considered best on day 1 with high sensitivity and good specificity, though with low positive predictive values (PPV) and high negative predictive values (NPV) for predicting candidemia. Low PPVs were reported by other studies when candida colonization parameters were used for predicting invasive candidiasis. This is attributed to the low prevalence of candidemia in paediatric lymphoreticular malignancy, hence the NPV can be used to rule out candida infection effectively [25,26]. ROC for colonization index day 1 shows 100% NPV at the cut-off of 0.4, which can be considered to rule out the risk of having candidemia as early as day 1 of fever which may assist in predicting candidemia at the time of admission and thus will help in directing further management.

In our study, mannan antigen detection had a sensitivity of 80% and specificity at 92.6%, a better indicator for candidemia compared to blood culture 4 (80%). On the contrary, a previous study observed the mannan diagnostic sensitivity at 77% and specificity at 51% [27]. A retrospective study reported mannan sensitivity to be 58% (95% CI: 53–62) and specificity 93% (95% CI: 91–94) [28]. They observed anti-mannan antibody sensitivity of 59% (95% CI: 54–65) and specificity of 83% (95% CI: 79–97) and a combined mannan/anti-mannan antibody sensitivity was 83% (95% CI: 79–87) and specificity 86% (95% CI: 82–90) was seen. The combination of both mannan and anti-mannan antibody detection tests detects candidemia with higher sensitivity and specificity. A positive mannan antigen test has been recorded several days before (median 6 days) in positive blood culture in a study by Prella et al. [29], similar to our observation in patients of candidemia. We assessed the diagnostic performance of mannan antigen level in predicting candidemia by ROC curve which revealed a cut-off of mannan antigen level ≥104.667 pg/mL, found suitable for predicting candidemia with a sensitivity of 100%, and a specificity of 92% and AUROC value of 0.958 (95% CI: 0.915–1; *p* < 0.001). Our study reported a higher sensitivity and specificity as compared to previous studies and supports the early detection of mannan antigen as a useful marker for candidemia compared to positive blood culture.

The in vitro antifungal susceptibility of *Candida* spp. was 100% sensitive to fluconazole, amphotericin B, and caspofungin, unlike the antifungal susceptibility of candidemia isolates at a multispecialty centre in North India which reported a 55–59% resistance to the azole group of antifungals [30]. The antifungal susceptibility profile in children with candidemia from a tertiary hospital in Delhi reported an 88.30% isolation of non-albicans candida from blood isolates, of which all the isolates had good activity against caspofungin [31]. The geographical distribution of Candida species, host profile and the differing policy of usage of antifungal prophylaxis determines the resistance to antifungals. Our blood isolates were confirmed by genotypic tools and a phylogenetic tree was created with three population groups including Clade 1, 2, 3 consisting of one isolate of *C. auris*, two isolates of *C. tropicalis* and two isolates of *C. parapsilosis*, respectively.

In resource-poor settings, the study provides simple diagnostic markers to assess the outcome of children with lymphoreticular malignancy at high risk of acquiring invasive fungal diseases (IFDs). However, other fungal biomarkers such as galactomannan and 1,3-β-d-glucan (BDG), and molecular tools such as fungal PCR or T2Candida assay can aid in early diagnosis. Despite the criteria as established by the European Organization for Research and Treatment of Cancer/Mycoses Study Group (EORTC/MSG), limitations for a systematic approach to define IFDs still exist. Hence, the use of biomarkers in combination with other molecular tests can help to overcome the limitations in sensitivity and specificity of the diagnostic tools. Further, to strengthen and optimize the strategy for appropriate diagnosis, follow-up studies should be recommended. Paediatric-specific studies of the biomarkers alone and in combination are needed to define the limitations of each assay and to design the optimal multipronged diagnostic strategy.

This study, though, was limited by the small sample size and brief study period.

## 5. Conclusions

The root cause of candidemia with predominant non-albicans candida species in patients with lymphoreticular malignancy is possibly the endogenously colonizing candida species in the host. Antifungal prophylaxis decreases colonization and the risk of candidemia. As blood culture is time consuming and has limited sensitivity, there is a need for new biomarkers for early detection and establishing rational antifungal treatment. The traditional colonization index can predict candidemia, but threshold value needs to be explored in paediatric patients with lymphoreticular malignancies. The diagnosis of candidemia based on mannan antigen detection gives early results and has high negative predictive values. It can be combined with other biomarkers to increase sensitivity, specificity and PPV.

## Figures and Tables

**Table 1:** Association of various clinical parameters with candidemia.

Clinical Parameters	Candidemia Present (*n* = 5)	Candidemia Absent (*n* = 95)	*p*-Value
Fever Duration (Days)	4.60 ± 2.61	3.76 ± 1.52	0.522 ^2^
Pain Abdomen (Present) ***	4 (80.0%)	18 (18.9%)	0.008 ^1^
Cough/Respiratory Distress (Present)	2 (40.0%)	9 (9.5%)	0.092 ^1^
Loose Stool (Present)	1 (20.0%)	15 (15.8%)	1.000 ^1^
Headache/Vomiting/Neck Rigidity (Present)	2 (40.0%)	9 (9.5%)	0.092 ^1^
Haemoglobin (Day 1)	7.38 ± 0.53	7.88 ± 0.86	0.168 ^2^
TLC (Day 1)	1260.00 ± 320.94	1530.53 ± 474.92	0.207 ^2^
ANC (Day 1)	420.00 ± 130.38	578.95 ± 184.47	0.061 ^2^
Haemoglobin (Day 4)	7.48 ± 0.59	8.04 ± 0.76	0.096 ^2^
TLC (Day 4) ***	1160.00 ± 288.10	1641.05 ± 423.89	0.014 ^2^
ANC (Day 4) ***	460.00 ± 89.44	713.05 ± 169.94	0.003 ^2^

*** Significant at *p* < 0.05, ^1^: Fisher’s exact test, ^2^: Wilcoxon-Mann-Whitney U test.

**Table 2:** Association of candida colonization and candidemia among febrile cases.

Risk Factor	Candidemia Present (*n* = 5)	Candidemia Absent (*n* = 95)	Odds Ratio (95%CI)	*p*-Value
Colonization Index ≥ 0.5 (Day 1) ***	4 (80%)	1 (1.05%)	376 (19.74–7161.52)	<0.001 ^1^
Colonization Index ≥ 0.5 (Day 4) ***	3 (60%)	1 (1.05%)	141 (9.85–2018.79)	<0.001 ^1^
Colonization Index ≥ 0.5 (Day 8)	1 (20%)	0 (0%)	63.67 (2.26–1791.83)	0.050 ^1^

*** Significant at *p* < 0.05, ^1^: Fisher’s exact test.

**Table 3:** Candida colonization at different anatomical sites on day 1, 4 and 8.

Mycological Surveillance	Candida Colonization (Day 1)	Candida Colonization (Day 4)	Candida Colonization (Day 8)
Sputum/Pharyngeal Swab	18%	15%	4.04%
Umbilical Swab	2%	1%	0%
Axillary Swab	1%	1%	0%
Rectal Swab	14%	6%	2.02%
Urine	3%	3%	2.02%

**Table 4:** Comparison of mannan antigen and colonization index as indicators of candidemia.

Predictor	AUROC	95% CI	Sn	Sp	DA	*p*-Value
Mannan Antigen Level	0.958	0.915–1	100%	92%	92%	0.001
Colonization Index (Day 1)	0.983	0.961–1	100%	94%	94%	<0.001
Colonization Index (Day 4)	0.975	0.941–1	100%	87%	88%	<0.001
Colonization Index (Day 8)	0.686	0.443–0.929	40%	97%	94%	<0.001

AUROC: area under ROC curve; CI: confidence interval; Sn: sensitivity; Sp: specificity; DA: diagnostic accuracy.

**Table 5:** Primary diagnostic parameters.

Variable	Sensitivity	Specificity	PPV	NPV	DA
Mannan Antigen Level (Cut-off: 104.667 by ROC)	80.0% (28–99)	91.6% (84–96)	33.3% (10–65)	98.9% (94–100)	91.0% (84–96)
Mannan Antigen	80.0% (28–99)	92.6% (85–97)	36.4% (11–69)	98.9% (94–100)	92.0% (85–96)
Colonization Index Day 1 (Cut-off: 0.4 by ROC)	100.0% (48–100)	93.7% (87–98)	45.5% (17–77)	100.0% (96–100)	94.0% (87–98)
Colonization Index Category (Day 1)	80.0% (28–99)	98.9% (94–100)	80.0% (28–99)	98.9% (94–100)	98.0% (93–100)
Colonization Index Day 4 (Cut-off: 0.2 by ROC)	100.0% (48–100)	87.4% (79–93)	29.4% (10–56)	100.0% (96–100)	88.0% (80–94)
Colonization Index Category (Day 4)	60.0% (15–95)	98.9% (94–100)	75.0% (19–99)	97.9% (93–100)	97.0% (91–99)
Colonization Index Day 8 (Cut-off: 0.2 by ROC)	40.0% (5–85)	96.8% (91–99)	40.0% (5–85)	96.8% (91–99)	94.0% (87–98)
Colonization Index Category (Day 8)	20.0% (1–72)	100.0% (96–100)	100.0% (3–100)	96.0% (90–99)	96.0% (90–99)

PPV: positive predictive values; NPV: negative predictive values; DA: diagnostic accuracy; ROC: receiver operating characteristics analysis.

**Table 6:** Association of candidemia with candida mannan antigen detection.

Variable	LR+	LR-	Youden’s Index	Odds Ratio	Kappa	*p*-Value
Mannan Antigen Level (Cut-off: 104.667 by ROC)	9.50 (4.29–21.03)	0.22 (0.04–1.26)	71.6	43.50 (4.33–437.30)	0.43	<0.001
Mannan Antigen	10.86 (4.70–25.07)	0.22 (0.04–1.25)	72.6	50.29 (4.93–513.00)	0.46	<0.001
Colonization Index Day 1 (Cut-off: 0.4 by ROC)	15.83 (7.30–34.35)	0.00 (0.00–NaN)	93.7	Inf (NaN–Inf)	0.6	<0.001
Colonization Index Category (Day 1)	76.00 (10.30–560.60)	0.20 (0.04–1.17)	78.9	376.00 (19.74–7161.52)	0.79	<0.001
Colonization Index Day 4 (Cut-off: 0.2 by ROC)	7.92 (4.67–13.43)	0.00 (0.00–NaN)	87.4	Inf (NaN–Inf)	0.41	<0.001
Colonization Index Category (Day 4)	57.00 (7.14–454.81)	0.40 (0.14–1.18)	58.9	141.00 (9.85–2018.79)	0.65	<0.001
Colonization Index Day 8 (Cut-off: 0.2 by ROC)	12.67 (2.70–59.49)	0.62 (0.30–1.27)	36.8	20.44 (2.44–171.49)	0.37	<0.001
Colonization Index Category (Day 8)	Inf (NaN-Inf)	0.80 (0.52–1.24)	20.0	Inf (NaN–Inf)	0.32	<0.001

ROC: receiver operating characteristics analysis; LR+: likelihood ratio for positive test results; LR−: likelihood ratio for negative test results; NaN: not-a-number; Inf: infinity.

**Table 7:** Ranking of primary diagnostic parameters.

Variable	Sensitivity	Specificity	PPV	NPV
Mannan Antigen Level (Cut-off: 104.667 by ROC)	3	7	7	5
Mannan Antigen	3	6	6	4
Colonization Index Day 1 (Cut-off: 0.4 by ROC)	1	5	4	1
Colonization Index Category (Day 1)	3	2	2	3
Colonization Index Day 4 (Cut-off: 0.2 by ROC)	1	8	8	1
Colonization Index Category (Day 4)	6	2	3	6
Colonization Index Day 8 (Cut-off: 0.2 by ROC)	7	4	5	7
Colonization Index Category (Day 8)	8	1	1	8

PPV: positive predictive values; NPV: negative predictive values; ROC: receiver operating characteristics analysis.

## Data Availability

The data presented in this study are available on request from the corresponding author.
